# Retention Rate of Hydrophilic and Hydrophobic Resin-Based Sealant under Field Conditions: A Split-Mouth Randomized Controlled Clinical Trial

**DOI:** 10.1055/s-0043-1777052

**Published:** 2024-02-22

**Authors:** Araya Thetsanasalee, Siriruk Nakornchai, Varangkanar Jirarattanasopha

**Affiliations:** 1 Department of Pediatric Dentistry, Faculty of Dentistry, Mahidol University, Bangkok, Thailand

**Keywords:** field conditions, hydrophilic, hydrophobic, retention, resin, sealant

## Abstract

**Objectives** The aim of this study was to evaluate and compare the clinical retention rate between hydrophilic and hydrophobic resin-based sealant placed under field setting and related factors.

**Materials and Methods** Sixty-six children with 106 pairs of teeth in the same arch with matching International Caries Detection and Assessment System (ICDAS) scores ranging from 0 to 2 were recruited. This study was a split-mouth design with each tooth in the pair randomly assigned into either hydrophobic resin-based sealant group (Concise white sealant, 3M. EPSE, St. Paul, Minnesota, USA) or hydrophilic resin-based sealant group (UltraSeal XT hydro sealant, Ultradent Products, South Jordan, Utah, USA). A dental therapist performed all procedures in a field setting on a mobile dental unit with a mobile saliva ejector. The retention rate was evaluated by two calibrated dentists and classified as fully retained, partially retained, and total loss.

**Statistical Analysis** The outcomes were analyzed using McNemar's, chi-squared, and Fisher's exact test with a significance level of 0.05.

**Results** After 12 months, 65 children with 105 pairs of teeth remained in this study. At 8-month follow-up, fully retained, partially retained, and total loss of material were found at 82.9, 15.2, and 1.9% in the hydrophobic group and 70.5, 26.7, and 2.9% in the hydrophilic group, respectively. At the 12-month follow-up, the outcomes were reduced, respectively, to 80, 17.1, and 2.9% in the hydrophobic group and 68.6, 27.6, and 3.8% in the hydrophilic group. There was no significant difference between the two groups ( *p* > 0.05). Arch type was associated with the retention rate ( *p* < 0.05), whereas ICDAS scores showed no correlation ( *p* > 0.05).

**Conclusion** Both hydrophilic and hydrophobic resin-based sealant can be used under field conditions, with no significant difference in terms of retention rate.

## Introduction

Dental caries is the most globally pervasive disease affecting general health along with impairing the quality of life of billions worldwide. 1 The adequate use of fluoride and mechanical plaque control has reduced the dental caries prevalence. 1 However, these preventive regimens are less effective on the occlusal part of the tooth where the complex natural morphology, pits, and fissures easily accumulate food debris and dental plaque. 2

Sealant is typically a resin-based or glass ionomer-based material that flows into and seals pits and fissures. 2 Placing a sealant physically blocks cariogenic substances and bacterial build-up in these deep grooves. 2 Sealant has long been used and reduces the risk of developing new carious lesions by 76%. 2 Placing a sealant is effective on sound tooth surface and can be used on early noncavitated caries to inhibit its progression. 3

The clinical retention of sealant material has been used to evaluate its efficacy. 2 This is because the barricade between the caries-related factors and the protected surface is due to the tight sealing quality of the material. The first marketed sealants, which are resin-based materials, have the highest retention rates among sealant types. 4 However, this material is highly technique sensitive due to its hydrophobicity. 4 Therefore, in conditions where adequate moisture control cannot be achieved, the retention rate is reduced. 4 Partially erupted teeth with soft tissue impingement, uncooperative children, or those with special needs, and not having the appropriate moisture-control equipment can result in unsatisfactory sealant retention. 5

A novel resin-based sealant was fabricated by modifying the chemical part of the monomer, which converted the hydrophobic sealant into a moisture-friendly material. 6 This material has been commercially available but is not widely used clinically. *In vitro* studies have demonstrated that the hydrophilic sealant has physical properties similar to the hydrophobic resin-based sealant, for example, low viscosity, long resin tag formation, and good marginal integrity with low microleakage. 7 However, the clinical comparison results are disparate and limited in number. A study found a significantly higher retention rate for hydrophilic sealant compared with a hydrophobic sealant. 8 A study in 2013 found the retention rate to be similar compared with a hydrophobic sealant, but superior compared with a glass ionomer sealant. 6 Moreover, a study found that a hydrophilic sealant had a poorer retention performance and surface quality. 9 According to its physical properties, a hydrophilic sealant has the potential to overcome challenging clinical situations. This may be beneficial to the use of sealant in less-than-ideal situations, such as the school-based sealant programs that are commonly performed in a field setting that is a challenging clinical environment.

The aim of this study was to evaluate and compare the clinical retention rate between hydrophilic resin-based sealant and hydrophobic resin-based sealant placed in a field setting after 8 and 12 months. The associated factors related to material retention were also assessed.

## Materials and Methods

### Sample Size Estimation

The McNemar's test of equality of paired proportions was applied considering that the test is two-sided with a significance level of 0.05 and power of 80%. The proportion of hydrophilic sealant was calculated at 80% in a similar study by Prabakar et al. 8 The expected proportion of the hydrophobic resin-based sealant was 70% according to Tianviwat et al, which concluded a difference in the proportion of 10% between both groups. 10 The proportion of discordant pairs was calculated at 14%. The calculation revealed that a sample size of 96 pairs was required. By adding 10% as the possible drop-out rate, the sample size was finalized at 106 pairs.

### Inclusion and Exclusion Criteria

The inclusion criteria were healthy, cooperative 6 to 9-year-old participants with bilateral fully erupted permanent first molars with deep pits and fissures and the same carious condition. The included molars were sound to noncavitated distinct visual change in enamel lesions or graded as 0, 1, or 2 according to the International Caries Detection and Assessment System (ICDAS).

Patients who did not fully cooperate throughout the procedure on both teeth along with teeth with enamel defects, proximal carious lesions, or restorations were excluded from the study.

### Sample Recruitment

The samples were recruited from a list of over 298 early elementary students in three public schools who were already participating in the school-based sealant program. The dental check-up was performed at school by a dental nurse. Informed consent was provided by the parents of the eligible participants.

### Clinical Procedure

This study was a prospective, randomized controlled with a split-mouth design. It was conducted in a field setting. This study protocol was approved by an Institutional Review Board (COA.No.MU-DT/PY-IRB 2021/024.2202). This study was registered in the Clinical Trial Registry (identification number: TCTR20230412003).

A total of 106 pairs of teeth were included and one tooth in each pair was assigned into two groups, hydrophobic or hydrophilic sealant, by computer-generated randomization. Both materials were equally divided into left-side teeth and right-side teeth groups.

The randomization process was done again within each group to determine which side of the arch the operator began applying the sealants. One half of the group, 26 pairs, was assigned to have the operator start on the right side followed by the left side and vice versa.

The operator was a skilled dental nurse who routinely conducted the school-based sealant program in the area. The operator was blinded to the type of material and the order in which the different materials were placed. The participants and the examiners who assessed the outcomes were also blinded to this information. Due to the similar opaque, slightly yellowish-white color of both materials, before and after polymerization, were indistinguishable post-setting.

This study was performed in a field setting on a mobile dental unit with a mobile saliva ejector. The sealant applications were performed by a single operator with the same assistant at the same operatory. Every step of the procedure with a specific time length was recorded by a research assistant using a digital timer.

The tooth was cleaned with a slow-speed rubber cup and pumice. The pumice was washed out with a copious stream of water with a gentle probing action using a dental explorer along the grooves to remove any remaining debris. Cotton rolls were placed on the buccal and lingual vestibule of the lower tooth and the buccal vestibule for the upper tooth. The tooth was dried with the triple syringe for 10 seconds to remove excess water that might affect the concentration of the subsequent acid etching procedure. The dried tooth was applied with either 37% phosphoric acid (Scotchbond Etchant, 3M-ESPE, St. Paul, Minnesota, United States) or 35% phosphoric acid (Ultra-etch Etchant, Ultradent, South Jordan, Utah, United States) for 20 seconds and rinsed with water for 20 seconds. The isolation procedure was performed with a new set of cotton rolls and dried with a triple syringe for 10 seconds. A fully dry surface without any visible film of moisture was expected.

The material was handed to the operator according to the order of treatment. In Group 1 hydrophobic sealant, the tooth was applied with Concise white sealant (3M. EPSE, St. Paul, Minnesota, United States). In Group 2, hydrophilic sealant, the tooth was applied with UltraSeal XT hydro sealant (Ultradent Products, South Jordan, Utah, United States). Both types of sealants were polymerized using an LED light-curing unit (Bluephase Style Ivoclar Vivadent Schaan, Liechtenstein) for 20 seconds with the tip placed directly over the applied surface. A dental explorer was used to probe along the grooves to detect any voids or roughness of the surface and reapplied if needed. The occlusion was tested by having the participants bite on articulating paper and adjusted with a slow-speed polishing bur if needed. The participants rinsed their mouths after the procedure was completed.

### Recall and Evaluation

The participants were originally asked to come back for an evaluation at follow-up periods of 6 and 12 months. Due to the pandemic lockdown, the follow-up periods were shifted to 8 and 12 months. The examination was done using the same mobile unit and lighting in the field setting. The process began with having the patient thoroughly brush and rinse with 1% hydrogen peroxide per the protocol for reducing the risk of coronavirus disease 2019 transmission. Two dentists who were blinded to the types of sealants applied were recruited to examine the teeth. Calibration was done prior to the recall dates. Recalibration of the two dentists on the recall dates was done by re-examining 10 participants. The intrareliability within the same examiner was tested by re-examining 10% of the participants. The tactile and visual examinations were performed by inspecting the sealant appearance under mobile lighting and using a dental explorer to glide along the grooves. The sealant quality was graded according to Simonsen's criteria; fully retained, partial loss, and total loss sealant. 11

In the fully retained group, the tooth presented with sealant on all primary grooves and no need to reseal. In the partially retained group, the tooth retained some of the material that was detected by a ledged margin indicating a dislodged bulk of material or visual exposure of the previously sealed deep pits and fissures. In the total sealant loss group, no evidence of sealant was detectable on any pits and fissures of the tooth.

If at any time during the recall period the included teeth were found to need immediate care, such as a progressing carious lesion or traumatic injury, the patients were referred to receive the appropriate dental treatments. However, neither of these conditions was found throughout this study.

### Statistical Analysis

The intrareliability of each examiner was tested by re-examining 10% of the participants. The Kappa statistic was calculated and accepted at Kappa more than 0.7. The inter-reliability of the examiners was calibrated by re-examining 10 participants. The Kappa statistic was accepted at Kappa more than 0.7.

The demographic data of the participants comprising sex, age, tooth type, and the carious condition were collected as descriptive data. The outcomes were analyzed using SPSS Statistics for Windows, Version 28.0. (SPSS Inc., Chicago, Illinois, United States). The retention rate between groups was compared using McNemar's test. The correlation of sealant retention and arch type or ICDAS score were analyzed using chi-squared and Fisher's exact test. A *p* -value less than 0.05 was considered statistically significant.

## Results

**Fig. 1:**
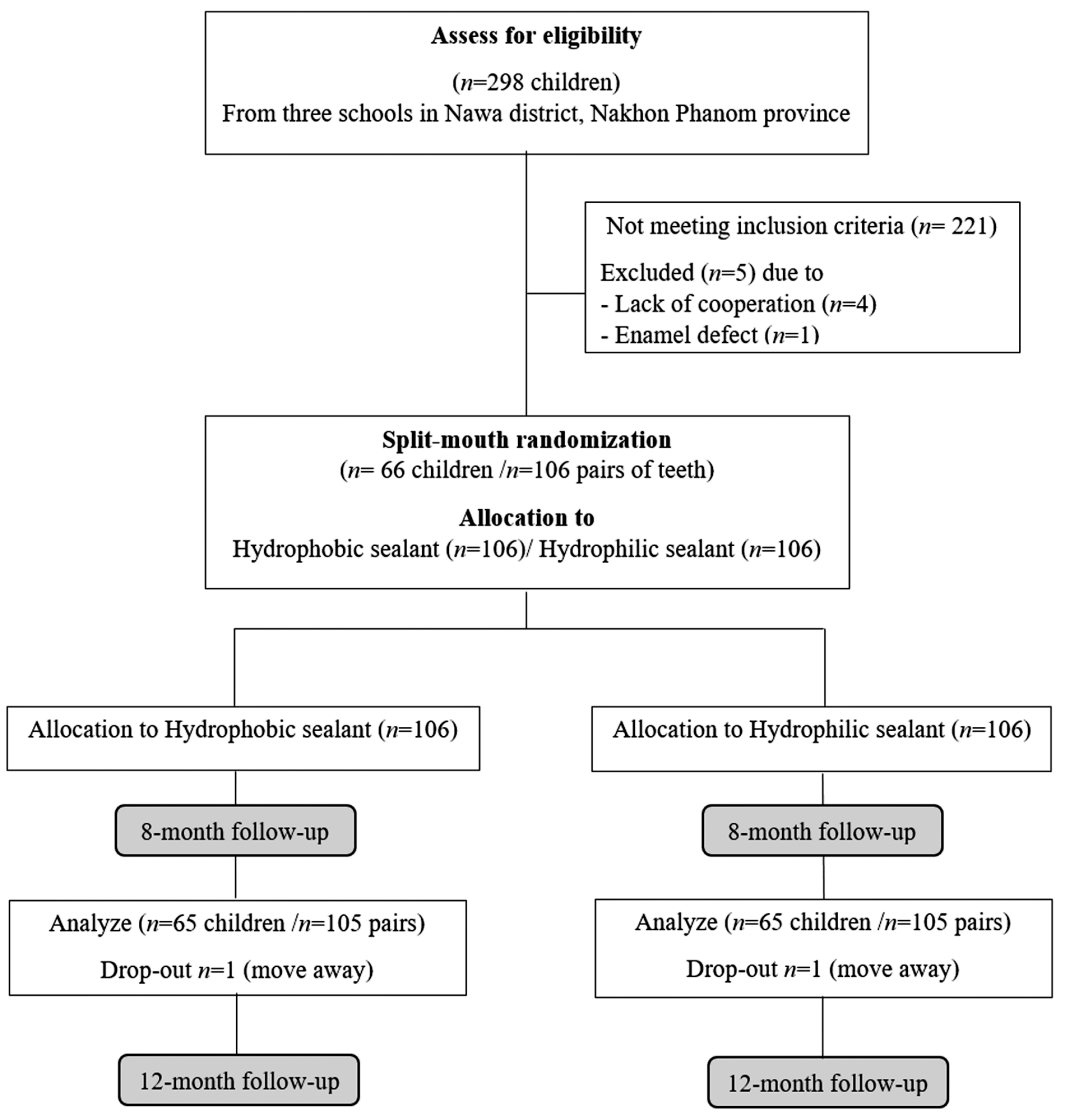
Study flow diagram.

Sixty-six children with 106 pairs of teeth were recruited according to the inclusion criteria. After 12 months of follow-up, one child dropped out due to relocation, thus, 65 children with 105 pairs of teeth completed this study. The 65 children (6–9 years old) comprised 36 males (55.4%) and 29 females (44.6%) with an average age of 7.51 years old. The flow of the participants in this study is presented in Fig. 1 . Sixty-one pairs of teeth were upper molars and 44 were lower molars. The type of tooth arch and ICDAS score are shown in Table 1 along with the equal distribution of materials on the left and right side of the arch.

**Table 1:** Distribution of hydrophobic and hydrophilic resin-based sealant by arch type, side, and ICDAS score

Group ( *n* = 105 pairs/210 teeth)	Arch type (teeth)		Right/left (teeth)		ICDAS score (teeth)		
	Upper	Lower	Right	Left	0	1	2
Hydrophobic sealant	61	44	52	53	50	31	24
Hydrophilic sealant	61	44	53	52	50	31	24
Total	122	88	110	110	100	62	48

Abbreviation: ICDAS, International Caries Detection and Assessment System.

The intra- and inter-reliability of both examiners were in good agreement with Kappa values of 0.84 and 0.81 for intra-reliability and 0.80 for inter-reliability.

The sealant retention after 8 and 12 months is demonstrated in Table 2 . At the 8-month follow-up, the sealant retention rate in each retention category between the two materials was not significantly different ( *p* = 0.051). For the 12-month follow-up, a slightly increased frequency was found in the partially retained and missing groups of both materials. However, there were no significant differences in the sealant retention rate of the two materials ( *p* = 0.140).

**Table 2:** The retention rate of hydrophobic and hydrophilic resin-based sealant at the 8- and 12-month follow-ups

Follow-up time (months)	Types of sealant	Retention, *n* (%)			*p* -Value
		Fully retained	Partially retained	Total loss	
8	Hydrophobic	87 (82.9)	16 (15.2)	2 (1.9)	0.051
	Hydrophilic	74 (70.5)	28 (26.7)	3 (2.9)	
12	Hydrophobic	84 (80)	18 (17.1)	3 (2.9)	0.140
	Hydrophilic	72 (68.6)	29 (27.6)	4 (3.8)	

McNemar's test.

The factors associated with the retention rates were distributed in Table 3 . In both groups, the materials applied in lower arch showed a superior retention rate compared with the materials on the upper arch ( *p* < 0.05). However, the difference in ICDAS scores showed no relationship with the retention rate in both groups.

**Table 3:** The correlation of sealant retention at 12-month follow-up between arch type and ICDAS score

Sealant type/factors		Fully retained, *n* (%)	Partially retained, *n* (%)	Total loss, *n* (%)	Total, *n* (%)	*p* -Value
Hydrophobic sealant	Upper	43 (70.5)	15 (24.6)	3 (4.9)	61 (100)	0.009
	Lower	41 (93.2)	3 (6.8)	0 (0.0)	44 (100)	
Hydrophilic sealant	Upper	35 (57.4)	22 (36.1)	4 (6.6)	61 (100)	0.007
	Lower	37 (84.1)	7 (15.9)	0 (0.0)	44 (100)	
Hydrophobic sealant	ICDAS 0	36 (72)	12 (24)	2 (4)	50 (100)	0.149
	ICDAS 1	29 (93.5)	2 (6.5)	0 (0.0)	31 (100)	
	ICDAS 2	19 (79.2)	4 (6.7)	1 (4.2)	24 (100)	
Hydrophilic sealant	ICDAS 0	32 (64)	14 (28)	4 (8)	50 (100)	0.360
	ICDAS 1	21 (67.7)	10 (32.3)	0 (0.0)	31 (100)	
	ICDAS 2	19 (79.2)	5 (20.8)	0 (0.0)	24 (100)	

Abbreviation: ICDAS, International Caries Detection and Assessment System.

Chi-squared and Fisher's exact test.

## Discussion

The objective of this study was to evaluate and compare the clinical retention rate between a hydrophilic resin-based sealant and hydrophobic resin-based sealant placed in a field setting after 8 and 12 months. This study was conducted in a field setting with a mobile dental unit. The field setting was chosen to demonstrate the actual dental treatment done in the school-based sealant program that has been performed in Thailand for decades. 10 12

The materials used in this study were UltraSeal XT hydro, which is commercially available and Concise white sealant, which is one of the oldest and widely used conventional resin-based sealants in Thailand's school-based sealant program. The hydrophilic resin sealant was developed to address the moisture-sensitive nature of the hydrophobic resin sealant. The hydrophobic bisphenol A-glycidyl methacrylate (bis-GMA) was replaced with di-, tri-, and multifunctional acidic acrylate monomers. This formulation creates a resin-based sealant that tolerates humidity and activates in the presence of moisture. 6

In our designed field settings, the retention rate in the hydrophobic group was lower than the expected performance of the hydrophobic resin-based sealant. The annual loss of conventional hydrophobic resin-based sealant was reported to be 5 to 10%. 13 However, the retention rate of this group was higher than a study by Tianviwat et al which reported that the average retention rate of sealant in Thailand's school-based sealant program was 20 to 63% at a 1-year recall. 10 The hydrophobic sealant group at the 12-month recall demonstrated a 72% fully retained rate, which aligned with a result of 73.1% from a study working in a clinical setting. 14

This study was conducted using a split-mouth design. The different types of materials were placed on two sides of the arch in the same patient. The advantage of this design is the exclusion of the interindividual variability effect on the outcomes. The carry-across effect, commonly found in the split-mouth design, did not apply to this study because the materials were placed and fixed on a specific site. Although one type of sealant used in this study released fluoride while the other did not, a study found that fluoride release did not affect sealant retention. 2 3 11

The recall period of this study was originally planned to be after 6 and 12 months. Studies indicated that sealant failure was mostly found immediately after application and at the 6-month follow-up. 9 11 However, due to the severe acute respiratory syndrome coronavirus 2 pandemic, the research location was under a long lockdown period. Access to the participants and research facility was not possible. Therefore, the recall period was extended to 8 months.

The field setting is a nonideal dental setting that can present problems during the critical steps of dental treatment. 12 Sealant placement, whose clinical success depends on moisture control, can be affected in this challenging setting. Moreover, the oral cavity is 100% humid by nature. Furthermore, the school-based sealant program was done in elementary school students whose total cooperation throughout the treatment cannot be expected. These factors are the possible reasons for unsatisfactory retention rates, along with the inadequate number of dental personnel. 12

This study used cotton rolls together with a saliva ejector and triple syringe as the moisture control procedure. The triple syringe was used for the same amount of time in both material groups. Although the desiccated etching pattern of enamel was observed after moisture control, the nonideal dental setting together with the humid nature of the oral cavity suggests that some moisture could be present within the fissures. These conditions favored the characteristic of hydrophilic sealant implying that higher retention rate of the sealant might be expected. However, the retention rate of hydrophilic and hydrophobic sealant is not significantly different. A similar field setting study with different form of moisture control, that is, removing water with cotton pallet to avoid desiccating the enamel, also found low retention rates of hydrophilic sealant with only 10% of fully retained material at the 12-month recall. 15 A possible explanation might be that the optimum level of moisture is inapplicable in practice. The manufacturer suggested that hydrophilic sealant works best on slightly moist teeth. However, the ideal level of moisture that optimizes the adhesion strength is challenging to replicate.

Sealants have the benefit of sealing and excluding the rough morphology of the pits and fissures from the factors leading to dental caries development and caries progression. Pit-and-fissure sealants provide primary prevention for sound teeth and secondary prevention for teeth in which signs of decalcification are present. The material's retention plays a critical role in clinical success because the protective barrier is dependent on the material binding to the tooth surface. Because the commonly used material is hydrophobic, moisture contamination jeopardizes sealant retention. Hydrophilic materials were formulated to overcome the moisture sensitive process of sealant placement and provide better retention. Our findings indicate that the hydrophilic resin-based sealant (UltraSeal XT hydro) is similar to the conventional hydrophobic resin-based sealant in maintaining the sealant on the tooth surface.

The retention rate of the materials on the lower arch group was significantly superior to the upper arch group. However, the different arch contribution to the retention rate of sealant is controversy. 16 17 This study conforms with a study by Ratnaditya et al that found higher retention rate of sealants on the lower arch. The higher retention found in lower arch may be from the easier access and direct visualized application of the sealant, the gravitation force; moreover, the better-defined pit and fissure anatomy of mandibular molars may also contribute to the outcomes. 17

This study included the molars with baseline surface status ranging from ICDAS 0 to 2, sound surface to the early visual change without enamel breakdown. The retention rate between different ICDAS scores demonstrated no significant difference after the 12-month recall. Another study found higher probability to fail in the early carious lesions, ICDAS 1 to 3, than the sound surfaces, ICDAS 0. 18 However, it might be influenced from the different inclusion criteria with another study's samples including ICDAS 3, the localized enamel breakdown without dentinal involvement. 18

Although the statistical analysis found no difference in the retention rates of the two material types, the clinical significance of the outcomes was notable. The conventional hydrophobic groups demonstrated higher retention rates compared with the hydrophilic sealant at both recall periods, 8 and 12 months. Because the exact amount moisture in favor of the hydrophilic sealant is still undetermined, replication of the profound moisture level may yield a different result. This may provide a better understanding of the material and indications for future research. Further studies are needed to develop an alternative moisture control strategy for pit and fissure sealant treatment.

## Conclusion

The hydrophilic resin-based sealant presented no significant different retention rate at the 8- and 12-month recalls compared with the hydrophobic resin-based sealant under field conditions.

## References

[1] Fact sheet: Oral Health. [Internet] Geneva World Health Organization 2020[updated 2020 May 25; cited 2020 September 20]. Accessed October 21, 2023 at:https://www.who.int/news-room/fact-sheets/detail/oral-health

[2] Wright J T Tampi M P Graham L Sealants for preventing and arresting pit-and-fissure occlusal caries in primary and permanent molars: a systematic review of randomized controlled trials-a report of the American Dental Association and the American Academy of Pediatric Dentistry J Am Dent Assoc 2016 147 08 631 6.45E20 27470524 10.1016/j.adaj.2016.06.003

[3] Wright J T Crall J J Fontana M Evidence-based clinical practice guideline for the use of pit-and-fissure sealants: a report of the American Dental Association and the American Academy of Pediatric Dentistry J Am Dent Assoc 2016 147 08 672 6.82E14 27470525 10.1016/j.adaj.2016.06.001

[4] Muller-Bolla M Lupi-Pégurier L Tardieu C Velly A M Antomarchi C Retention of resin-based pit and fissure sealants: a systematic review Community Dent Oral Epidemiol 2006 34 05 321 336 16948671 10.1111/j.1600-0528.2006.00319.x

[5] European Academy of Paediatric Dentistry Welbury R Raadal M Lygidakis N A EAPD guidelines for the use of pit and fissure sealants Eur J Paediatr Dent 2004 5 03 179 184 15471528

[6] Bhat P K Konde S Raj S N Kumar N C Moisture-tolerant resin-based sealant: a boon Contemp Clin Dent 2013 4 03 343 348 24124301 10.4103/0976-237X.118394 PMC3793556

[7] Prabhakar A R Murthy S A Sugandhan S Comparative evaluation of the length of resin tags, viscosity and microleakage of pit and fissure sealants - an in vitro scanning electron microscope study Contemp Clin Dent 2011 2 04 324 330 22346161 10.4103/0976-237X.91797 PMC3276861

[8] Prabakar J John J Arumugham I M Kumar R P Srisakthi D Comparative evaluation of retention, cariostatic effect and discoloration of conventional and hydrophilic sealants - a single blinded randomized split mouth clinical trial Contemp Clin Dent 2018 9(2, Suppl 2):S233 S239 30294150 10.4103/ccd.ccd_132_18 PMC6169278

[9] Schlueter N Klimek J Ganss C Efficacy of a moisture-tolerant material for fissure sealing: a prospective randomised clinical trial Clin Oral Investig 2013 17 03 711 716 10.1007/s00784-012-0740-2 22552593

[10] Tianviwat S Hintao J Chongsuvivatwong V Thitasomakul S Sirisakulveroj B Factors related to short-term retention of sealant in permanent molar teeth provided in the school mobile dental clinic, Songkhla province, Southern Thailand J Public Health 2011 41 01 50 58

[11] Simonsen R J Neal R C A review of the clinical application and performance of pit and fissure sealants Aust Dent J 2011 56(1, Suppl 1):45 58 21564115 10.1111/j.1834-7819.2010.01295.x

[12] Hintao J Tianviwat S Retention rate and methods for improving pit and fissure sealant programs in Thailand J Public Health Dent 2013 11 01 47 61

[13] Feigal R J Sealants and preventive restorations: review of effectiveness and clinical changes for improvement Pediatr Dent 1998 20 02 85 92 9566011

[14] Haricharan P B Voruganti S Kotha A Mahalakshmamma Shivanna M Gandhi B Suresh N An efficacy study between high viscosity glass ionomers and resin sealants in fissure caries prevention: a 2-year split mouth randomized controlled trial Eur J Dent 2022 16 01 137 144 34433220 10.1055/s-0041-1731925 PMC8890911

[15] Haricharan P B Barad N Patil C R Voruganti S Mudrakola D P Turagam N Dawn of a new age fissure sealant? A study evaluating the clinical performance of embrace WetBond and ART sealants: results from a randomized controlled clinical trial Eur J Dent 2019 13 04 503 509 31891967 10.1055/s-0039-1696894 PMC6938448

[16] Mohapatra S Prabakar J Indiran M A Kumar R P Sakthi D S Comparison and evaluation of the retention, cariostatic effect, and discoloration of conventional Clinpro 3M ESPE and Hydrophilic Ultraseal XT Hydro among 12-15-year-old schoolchildren for a period of 6 months: a single-blind randomized clinical trial Int J Clin Pediatr Dent 2020 13 06 688 693 33976497 10.5005/jp-journals-10005-1859 PMC8060934

[17] Ratnaditya A Kumar M Sai S Zabirunnisa M Kandregula C Kopuri R Clinical evaluation of retention in hydrophobic and hydrophilic pit and fissure sealants-a two year follow-up study J Young Pharm 2015 7 171 179

[18] Berdouses E D Michalaki M Tsinidou K Vlachou A Pantazis N Oulis C J Effectiveness of fissure sealants on initial caries lesions (ICDAS 1-3) of permanent molars: a 4-year follow-up Eur J Paediatr Dent 2021 22 03 180 188 34544245 10.23804/ejpd.2021.22.03.2

